# Somatostatin signaling via SSTR1 contributes to the quiescence of colon cancer stem cells

**DOI:** 10.1186/s12885-016-2969-7

**Published:** 2016-12-07

**Authors:** Shirin R. Modarai, Lynn M. Opdenaker, Vignesh Viswanathan, Jeremy Z. Fields, Bruce M. Boman

**Affiliations:** 1 Department of Biological Sciences, University of Delaware, 118 Wolf Hall, Newark, DE 19716 USA; 2 Center for Translational Cancer Research, Helen F. Graham Cancer Center and Research Institute, 4701 Ogletown-Stanton Rd, Newark, DE 19713 USA; 3 CATX Inc., Gladwyne, PA 19035 USA

## Abstract

**Background:**

Neuroendocrine cells (NECs) reside adjacent to colonic stem cells (SCs) in the crypt stem cell (SC) niche, but how NECs are involved in regulation of SCs is unclear. We investigated NECs expressing somatostatin (SST) and somatostatin receptor type 1 (SSTR1) because SST inhibits intestinal proliferation. Hypothesis: SSTR1 cells maintain SCs in a quiescent state, and aberrant SST signaling contributes to SC overpopulation in colorectal cancer (CRC).

**Methods:**

The proportion of SCs to NECs cells was quantified, by flow cytometry, in CRC cell lines and primary normal/tumor tissues based on cellular ALDH and SSTR1 levels, respectively. Doubling time and sphere-formation was used to evaluate cell proliferation and stemness. CRC cell lines were treated with exogenous SST and SST inhibitor cyclosomatostatin (cycloSST) and analyzed for changes in SCs and growth rate. Paracrine signaling between NECs and SCs was ascertained using transwell cultures of ALDH+ and SSTR1+ cells.

**Results:**

In CRC cell lines, the proportion of ALDH+ cells inversely correlates with proportion of SSTR1+ cells and with rate of proliferation and sphere-formation. While primary normal tissue shows SST and SSTR1 expression, CRC shows only SSTR1 expression. Moreover, ALDH+ cells did not show SST or SSTR1 expression. Exogenous SST suppressed proliferation but not ALDH+ population size or viability. Inhibition of SSTR1 signaling, via cycloSST treatment, decreased cell proliferation, ALDH+ cell population size and sphere-formation. When co-cultured with SSTR1+ cells, sphere-formation and cell proliferation of ALDH+ cells was inhibited.

**Conclusion:**

That each CRC cell line has a unique ALDH+/SSTR1+ ratio which correlates with its growth dynamics, suggests feedback mechanisms exist between SCs and NECs that contribute to regulation of SCs. The growth suppression by both SST and cycloSST treatments suggests that SST signaling modulates this feedback mechanism. The ability of SSTR1+ cells to decrease sphere formation and proliferation of ALDH+ cells in transwell cultures indicates that the ALDH subpopulation is regulated by SSTR1 via a paracrine mechanism. Since ALDH+ cells lack SST and SSTR1 expression, we conjecture that SST signaling controls the rate of NEC maturation as SCs mature along the NEC lineage, which contributes to quiescence of SCs and inhibition of proliferation.

**Electronic supplementary material:**

The online version of this article (doi:10.1186/s12885-016-2969-7) contains supplementary material, which is available to authorized users.

## Background

In colorectal cancer (CRC) development, the overpopulation of neoplastic stem cells (SCs) appears to drive tumor initiation and progression, but it is not really known which specific mechanisms that regulate normal colonic SCs, when dysregulated, result in SC overpopulation in CRC [1–4]. We surmised that the interactions and communication between different cell types within the colonic crypt SC niche may be crucial to regulation of normal SCs. Specific types of neuroendocrine cells (NECs), such as somatostatin receptor 1 cells (SSTR1), have been shown to reside in close proximity to colonic SCs in the niche at the bottom of the normal human colonic crypt (see Additional file 1: Figure S1). NECs are known to function in inhibition and/or enhancement of cell proliferation either by paracrine or autocrine signaling [5–8]. Nonetheless, the mechanisms through which SCs and specific NECs interact with each other in the normal colon have not been extensively studied. We hypothesize that SSTR1 cells maintain colonic SCs in a quiescent state, and aberrant SST signaling contributes to SC overpopulation in CRC.

Indeed, a substantial body of evidence reveals that various types of NECs are located along the normal intestinal tract and each NEC subtype has a different effect on neighboring cells [6, 7, 9, 10]. Specific NEC functions include secretion of peptides to act in a paracrine or autocrine fashion to exert local effects on cell proliferation and differentiation, or exert distant effects by endocrine secretion [7]. These NECs are often selectively located within the SC niche where the colonic SCs reside in a quiescent state. Thus, the niche likely provides the cues underlying slow-cycling dynamics of the SC population and asymmetric SC division that maintains the hierarchical nature of differentiated cell lineages in the colonic crypt [2]. Of note, colonic NECs do not appear to follow the classical hierarchical model of SC differentiation and are thought to arise by direct differentiation of a colonic SC, again supporting the close interactions between the two cell types [8]. Consequently, it seems feasible that the communication between NECs and colonic SCs is crucial to normal crypt homeostasis and maintenance of the quiescent nature of colonic SCs, and that dysregulation of the interactions and communication between the cell types could lead to colonic SC overpopulation during CRC progression.

To investigate possible regulatory mechanisms it must be technically feasible to identify, track and isolate human colonic SCs. Among various SC markers available, we have found that aldehyde dehydrogenase (ALDH) serves as a reliable and specific marker for normal and malignant colonic SCs [11–14]. Consequently, we used the ALDEFLUOR assay in this study to identify the ALDH+ colonic SCs and CSCs populations. For example, our previous study showed that ALDH+ cells are localized to the bottom of the colonic crypt and during progression from normal to adenoma, the number of ALDH+ cells increases supporting the concept that SC overpopulation leads to colon tumor development [11]. In our study [11] other SC markers such as CD133 and CD44 were compared to ALDH and we found that ALDH is the most specific marker for identification of human colon SCs [11]. In our current study we also used *in vitro* approaches including transwell co-cultures to ascertain the effects of SSTR1 signaling on ALDH+ cells. Our goal was to investigate how SSTR1 cell signaling contributes to changes to the ALDH+ cell population size and whether or not this regulation is by a paracrine mechanism. To our knowledge, this is the first attempt of looking at the effects of SSTR1 cell signaling directly on the ALDH+ population.

## Methods

### Cell culture

**Fig. 1:**
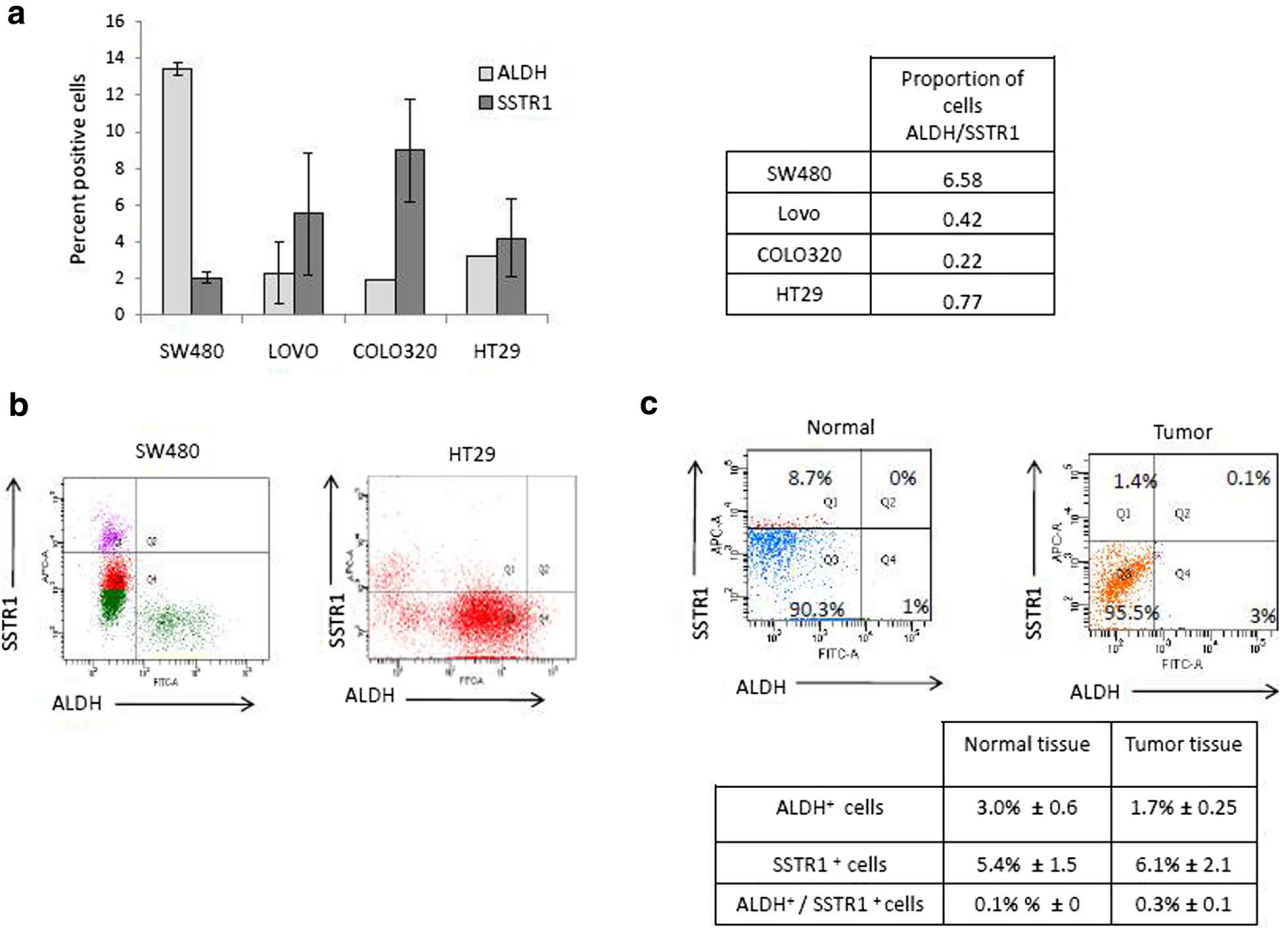
Differential expression pattern of ALDH and SSTR1 in colon cancer cell lines and patient tissue samples identifies unique populations of cells. **a** Percent positive ALDH and SSTR1 cells in various colon cancer cell lines. ALDH analysis was performed using ALDEFLUOR assay, and SSTR1 cell surface staining was determined against the appropriate IgM control. The proportions of ALDH/SSTR1 positive cells is shown in the table. Data represents mean ± S.E.M. (*N* = 3). **b** Representative dot plots of SSTR1 and ALDH expression analysis of SW480 cells and HT29 cells. **c** Representative dot plots of one matched patient colon normal and tumor tissue sample to show the expression analysis of ALDH and SSTR1 cells. Matched normal and tumor tissue samples were obtained and processed as described in the Materials and Methods section. All samples were run on the FACSAria II Flow Cytometer. Q = quadrant number, FITC channel detects ALDH positive cells using ALDEFLUOR assay, and APC channel detects SSTR1 positive The values in the table are averages ± SD; *N* = 4 matched patient samples

HT29 cells obtained from American Type Culture Collection (ATCC; Manassas, VA) were grown in monolayer cultures and maintained in: McCoys medium (GIBCO/Life Technologies) supplemented with 5% fetal bovine serum (FBS) and 100 units/ml penicillin and 100ug/ml streptomycin (P/S). SW480 cells obtained from ATCC were maintained in Leibovitz’s 15 (L-15) medium (GIBCO/Life Technologies) supplemented with 5% FBS and P/S. LoVo, DiFi and COLO320 cells were maintained in Roswell Park Memorial Institute (RPMI-1640) medium (GIBCO/Life Technologies) supplemented with 5% FBS and P/S. LoVo and DiFi cells were grown in monolayer cultures, while COLO320 cells were grown in suspension. DiFi and COLO320 cells were used as controls to verify NE cell marker expression and slow cell growth, as these cell lines contain high levels of NE positive cells [15, 16]. Since DiFi cells grow in monolayer cultures, this cell line was used in Fig. 3 for comparison to the other CRC cell lines for cell proliferation, and the COLO320 cells were used in Fig. 1 for positive identification of NE markers. COLO320 cells are reported to express high percentages of cells that secrete NE-like factors like serotonin, PTH, ACTH and other polypeptide hormones that are characteristics common to NE cells [15]. All cell cultures were maintained at 37°C in humidified air at 5% CO_2_.

### ALDEFLUOR assay

Protocol was followed according to the manufacturer (STEMCELL Technologies). Briefly, cells were grown to 80% confluence and lifted using 0.25% Trypsin-EDTA (Fisher Scientific). Cells were resuspended in ALDEFLUOR assay buffer at a concentration of one million cells/ml; to the control tube, 5 μl of the DEAB inhibitor was added and to the sample tube, 5 μl of the activated ALDEFLUOR reagent was added, mixed and immediately 500 μl of the suspension was taken out and put in the control tube with the inhibitor. Cells were incubated for 40 min at 37° C. After incubation, cells were spun for five minutes to pellet and washed once with ALDEFLUOR buffer. Cell were resuspended in 500 μl ALDEFLUOR buffer and passed through a BD round bottom tube with a 50 μm cell strainer (BD Biosciences). Samples were placed on ice and covered from light until ready for analysis on the BD FACSAria II Flow Cytometer.

### Flow cytometry

All cells were grown to 70–80% confluence and lifted using an EDTA based solution called Cell Stripper (Fisher Scientific). Cells were spun for five minutes to pellet and resuspended in RPMI-1640 with either the SSTR1 antibody (Advanced Targeting System) at a 1: 100 dilution or rabbit IgM control at an equal concentration to the antibody. Cells were incubated on ice for one hour. Following primary antibody and IgM incubation, cells were washed twice with PBS, and then incubated in the appropriate secondary antibody at a concentration of 1:200 for one hour on ice. Cells were washed twice and resuspended in 500 μl of RPMI-1640 medium and passed through a BD round bottom tube with a 50 μm cell strainer (BD Biosciences). Cell surface staining was analyzed using the BD FACSCalibur and BD FACSAria II Flow Cytometer. For our co-staining analysis, we chose to use FITC and APC as our fluorophores because the spectral overlap between these two fluorophores is minimal [17].

## Crypt isolation from normal colon tissue and tumor colon tissue dissociation

### Human tissues approval

Our research involving human colonic tissue was performed in accordance with the Declaration of Helsinki and was approved by the appropriate Institutional Review Board (FWA00006557) at Christiana Care Health Services, Inc (Newark, DE).

### Normal tissue

Crypt Isolation –The mucosa layer was dissected from the tissue and excess connective tissue, muscle and fat were trimmed off. Mucosa layer was washed with PBS (calcium and magnesium free) three times and then incubate tissue in 3 mM EDTA (pH = 8) that has 0.5 mM dithiothreitol (DTT) for 30 min on ice. After 30 min, the tube was shaken vigorously and checked for crypts. Crypts were collected in a separate tube and tissue was further incubated with fresh EDTA-DTT solution for another 30–60 min. Again, the tube was shaken vigorously for 5 min and isolated crypts were pooled in one tube. Crypts were spun down at 500 rpm for 5 min, washed two times with PBS, and pelleted again. Isolated crypts were incubated in 1 mg/ml collagenase IV (Worthington) in HBSS (Hanks Balanced Salt Solution) containing 100 units/ml DNase I for 60–90 min. The cell suspension was passed through 70 μm and 40 μm filters (Partec), and cells were re-spun. After washing twice with PBS, the final single cell suspension was analyzed using the ALDEFLUOR assay and Propidium Iodide (PI) at a dilution of 1:20. All cells were passed through a BD round bottom tube with a 50 μm cell strainer (BD Biosciences) and then a 20 μm filter (Partec) before samples were run on the BD FACSAria II Flow Cytometer.

### Tumor tissue

Tumor tissue was washed three times with PBS, minced into tiny 1mm pieces, and cut tissue was put in 1x collagenase/hyaluronidase (STEMCELL Technologies) and 100 units/ml DNaseI in HBSS and incubated for 60–90 min at 37° C. Cells were pelleted and passed through a 70 μm filter. Pelleted cells were incubated in red blood cell (RBC) buffer (NH_4_Cl, KHCO_3_, 5% EDTA solution) for 5 min on ice. Cells were washed twice with PBS and analyzed by ALDEFLUOR assay and APC conjugated EpCAM antibody at a dilution of 1:100 (Cell Signaling). All cells were passed through a BD round bottom tube with a 50 μm cell strainer (BD Biosciences) and then a 30 μm filter (Partec) before samples were run on the BD FACSAria II Flow Cytometer.

### Reverse transcriptase-polymerase chain reaction

RNA was collected from HT29 and SW480 cells when they were 80% confluent. RNA was harvested using the TRIzol method (Invitrogen). RNA was treated with DNaseI using the DNA-free DNA Removal Kit (Ambion) and the concentration of RNA was determined using the TECAN Infinite 200 PRO microplate reader. cDNA was created using the SuperScript III First-Strand Synthesis System (Life Technologies). Six sets of primers were used for the RT-PCR: somatostatin, SSTR1, SSTR2, SSTR3, SSTR4, and SSTR5. Primers were obtained from a previously published article [18]. For each sample 100ng of cDNA was used. Products were run on a 1.5% agarose gel with ethidium bromide and imaged using the Syngene imaging system.

### Cell proliferation

All cells were plated at a concentration of 20,000 cells/well of a 24 well plate (four wells per cell line). Medium was replaced every other day. Cells were detached using 0.25% Trypsin-EDTA at days 1, 3 and 5, and counted with a hemocytometer. Cells were mixed in a 1:1 ratio with Trypan blue (Fisher Scientific) so reported cell counts include only viable cells. This was repeated three times and the average values were graphed.

### Soft agar assay

Two percent agar and culture medium (depending on the cell line) containing 5% FBS were mixed in a 1:1 ratio, yielding a final concentration of 1% agar. This layer was poured into each well of a 24 well plate (Griener) and allowed to solidify. A second layer containing 0.25% agar in culture medium with 5,000 cells/well was poured over the first layer of agar and allowed to solidify. When the second layer solidified, culture medium was added to each well. Medium was changed every two days and cultures were allowed to grow for two weeks before being fixed and stained with 0.05% crystal violet and then visualized on a phase microscope.

### Somatostatin treatment

HT29 and SW480 cells were treated with somatostatin (500nM) (Tocris). This concentration was determined from the dose response curve (Additional file 2: Figure S2). Cells were plated at a concentration of 100,000 cells/well of a 6 well plate, in triplicate, and allowed to attach overnight. Cells were serum starved for 24 h in McCoy’s medium or L-15. Somatostatin was diluted in fresh medium and added to the cells for 48 h following serum starvation unless stated otherwise. All cells received somatostatin and in the corresponding control cells, an equal volume of vehicle was added.

### Cyclosomatostatin treatment

HT29 and SW480 cells were treated with 10 uM cyclosomatostatin (Tocris), which is within the accepted concentration range used to inhibit SST signaling without affecting cell viability [19]. Cells were plated at a concentration of 100,000 cells/per well of a 6 well plate, in triplicate, and allowed to attach overnight. Cells were serum starved for 24 h in McCoy’s medium (HT29) or L-15 medium (SW480). Cyclosomatostatin was diluted in fresh medium and added to the cells for 48 h following serum starvation. All experimental cells received cyclosomatostatin and the corresponding control cells, an equal volume of vehicle (culture medium) was added.

### Colonosphere assay

Cells were plated at a cell density of 200 cells per 100 μl of stem cell media which is composed of serum free DMEM/F12 (GIBCO Inc.) with the addition of Epidermal Growth Factor (EGF) and basic Fibroblast Growth Factor (bFGF) and B-27 complex without Vitamin A (Life Technologies, Carlsbad, CA). The method and culture medium used to perform the colonosphere assay was from a previously published article [20]. Low attachment plates were used for this assay and colonospheres were analyzed for their size (diameter) and numbers per well on day ten using the 10x objective of a phase contrast microscope.

### Co-culture of ALDH+ cells with SSTR1+ cells in culture dishes

To evaluate the effect of SSTR1+ cell signaling on ALDH+ cells, both of these cell subtypes were sorted from HT29 and SW480 cells and co-cultured in ultra-low attachment dishes with transwell inserts (0.4 um). The use of culture inserts allowed for the ALDH+ and SSTR1+ cells to be co-cultured without having cell-cell contact in order to study paracrine signaling. After ALDH+ and SSTR1+ cells were sorted on the BD FACSAria II Flow Cytometer, they were plated under different culture conditions. ALDH+ cells were plated in the bottom chamber with or without SSTR1+ cells in the top chamber and with or without the addition of exogenous SST. The effect of SSTR1+ cell signaling on ALDH+ cells was assessed by sphere formation.

### Statistics

All statistics were performed using Student’s t-test using Microsoft excel or one- way ANOVA using Graph Pad Prism software analysis.

## Results

### Quantification of ALDH+ and SSTR1+ cells in CRC cell lines

Five different human CRC cell lines were screened and each line showed a unique proportion of ALDH+ and SSTR1+ cells that was constantly maintained over time and upon multiple passages (Fig. 1a and Additional file 3: Figure S3). SW480 had the highest percent of ALDH+ cells, while the HT29 cell line had the lowest percentage. The COLO320 cell line contained the highest percentage of SSTR1+ cells, and HT29 and LoVo had the next highest percent of SSTR1+ cells. Based on these results, we used the ALDH+ to SSTR1+ quotient as a marker and measurable variable for the different CRC cell lines (Fig. 1a). The CRC cell line (SW480) had a high ratio value (~7.0) and three lines (LoVo, COLO320 & HT29) had low values (<1.0). Thus, based on the proportion of NECs, the relative proportion of CSCs varies among the different cell lines analyzed. These findings suggest that the proportion of SSTR1+ is inversely correlated to the proportion of ALDH+ cells.

To further characterize the subpopulations of ALDH+ and SSTR1+ cells, several other tests were performed on SW480 and HT29 lines. First, we wanted to rule out the possibility that some ALDH+ cells might also express SSTR1. Using double flow cytometric selection employing ALDEFLUOR assay with SSTR1 immunostaining, we found that the percentage of cells that co-stained for ALDH and SSTR1 was minimal. For example, the SW480 and HT29 cell lines showed that only 0.1% of cells co-expressed ALDH and SSTR1 (Fig. 1b).

### Correlation between CRC cell lines and matched human normal & malignant colon tissue

To see how the proportions of SSTR1 and ALDH in CRC cell lines compare to fresh human CRCs, matching normal and tumor colon tissue samples were collected from surgery patients. Tissues were then dissociated into single cells and analyzed by flow cytometry for ALDH and SSTR1. Figure 1c shows a representative histogram of a matched colon normal and tumor tissue sample pair to show the percentages of ALDH+ and SSTR1+ cells. Results on matched human patient sample pairs showed that the proportions of ALDH+ and SSTR1+ cells were in the same range of values as found for the CRC cell lines (Fig. 1b-c). Human colon tissues were also assessed for the proportion of cells that co-express ALDH and SSTR1 and the same low percentage of co-staining cells was seen as was observed in the CRC cell lines (Fig. 1c).

## Expression of somatostatin signaling components in CRC cell lines and fresh human colon tissues

**Fig. 2:**
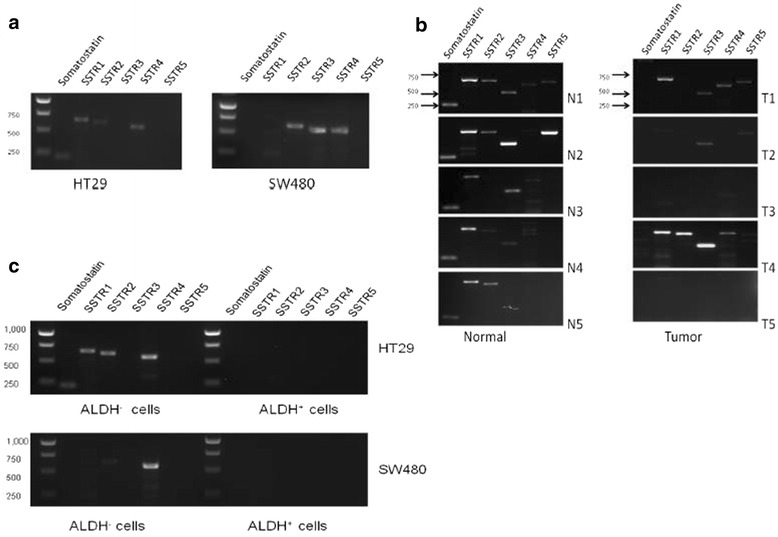
Expression of Somatostatin and its receptors in HT29 and SW480 colon cancer cell lines and patient matched normal and tumor tissue samples. **a** Cells were grown to 70–80% confluency, RNA was isolated and RT-PCR was performed. The HT29 cells express SST, SSTR1, SSTR2, and SSTR4. The SW480 cells express faint SSTR1, SSTR2, SSTR3, and SSTR4. This analysis was repeated three times and the gel images are representative of one data set for each cell line. **b** RT-PCR was performed to look at mRNA levels of SST and SSTR1-5 in these 5 patient samples. In all the normal samples, SST and SSTR1 were expressed. However, in the matching tumor samples, SST expression was gone and SSTR1 was present in 4 out of 5samples. N1 and T1 = matched normal and tumor tissue of patient #1. **c** Sorted ALDH+ and ALDH- cells from SW480 and HT29 cell lines were analyzed for mRNA expression of somatostatin and its receptors. RT-PCR analysis was performed on ALDH- and ALDH+ cells. These results indicate that the ALDH+ cells do not co-express somatostatin or its receptors in either cell line

We determined, by conventional RT-PCR, if somatostatin (SST) and its receptors (SSTR1-5) are expressed in SW480 and HT29 cells as well as in matched fresh normal and tumor human colon tissue samples. Results on CRC lines showed that both HT29 and SW480 cells express SST, SSTR1, SSTR2, and SSTR4, but the transcript level is more abundant in HT29 cells than SW480 cells (Fig. 2a). These results on SSTR1 transcript level concur with the encoded protein receptor levels.

Samples were also collected from patients and tissues were dissociated into single cells. RNA was isolated from the samples and the mRNA expression of SST and its receptors was analyzed using conventional RT-PCR. In matched fresh colon samples, a notable difference was observed in expression of somatostatin between normal and cancer. While all normal samples expressed SST, SST expression was absent in all matched tumor samples. SSTR1 was present in all normal samples and in 3 out of 5 tumor samples (Fig. 2b). Results were comparable between the CRC cell lines and analysis on human patient CRC samples (Fig. 2a-b).

We then determined, by RT-PCR, if somatostatin and its receptors are expressed in ALDH+ and ALDH- cells isolated from HT29 and SW480 lines. ALDH- cells from the HT29 line expressed SST, SSTR1, SSTR2, and SSTR4, while the sorted ALDH+ cells did not express SST or any of its receptors (Fig. 2c). Similarly, ALDH- cells from SW480 expressed SSTR1 (low), SSTR2, and SSTR4, while the ALDH+ cells did not express SST or any of its receptors (Fig. 2c). Overall, these findings suggest that at the mRNA and protein level, there is no appreciable expression of SST or SSTR1 in the ALDH+ cells from either HT29 or SW480 CRC cell lines.

## Proliferation rate inversely correlates with ALDH/SSTR1 quotient for CRC cell lines

**Fig. 3:**
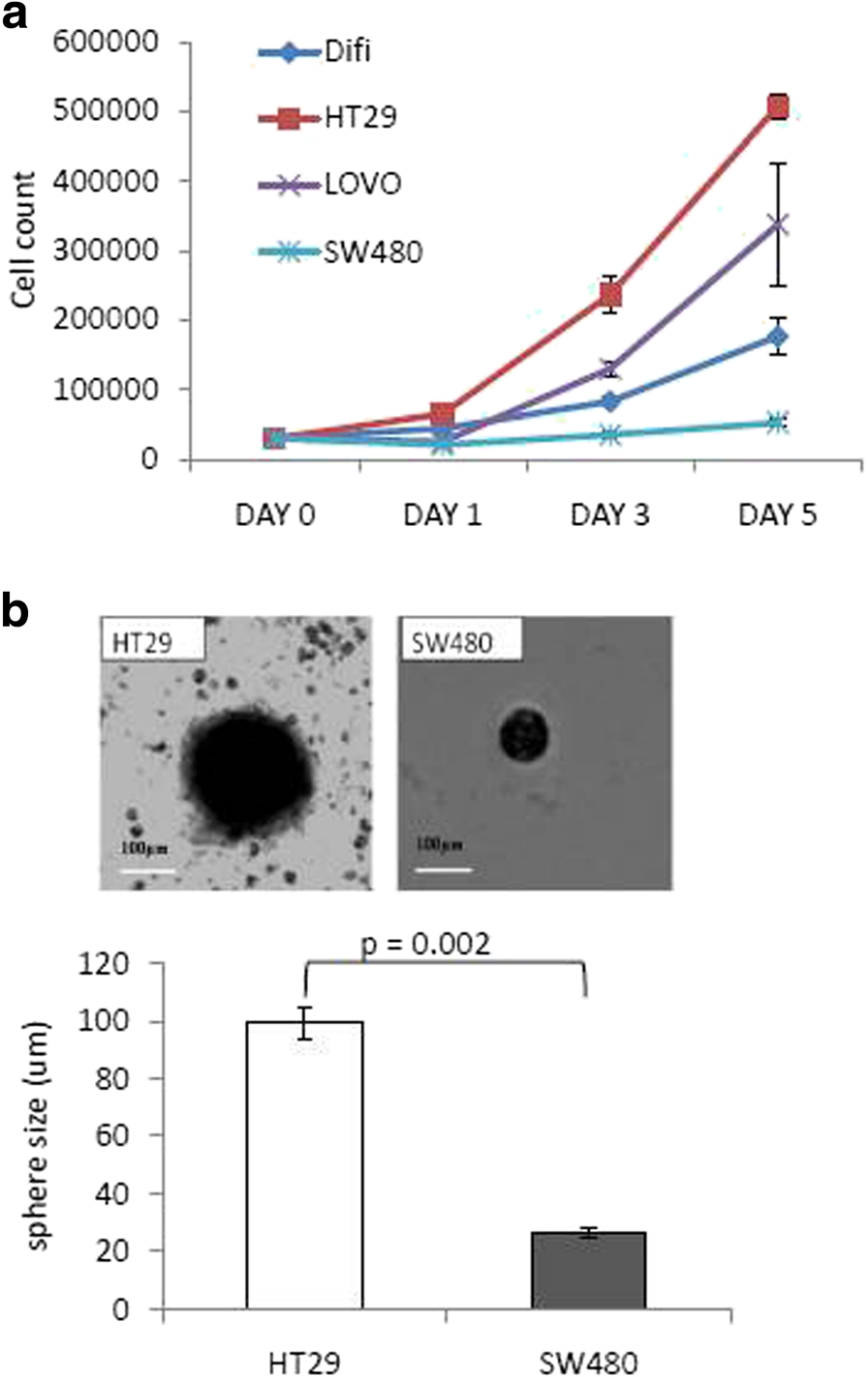
Increased proliferation rate and self-renewal ability of HT29 cells over SW480 cells. **a** Growth of colon cancer cell lines under normal culture conditions. An equal number of cells per well were plated and counted at days 0, 1, 3 and 5 with a hemocytometer. Dead cells were excluded by trypan blue analysis. Data represent mean S.E.M. (*N* = 3). **b** These images represent a sphere formed from a single cell, after two weeks in culture. Both images were taken under bright field with a 10x objective lens, on the Zeiss Epi-Fluorescence microscope. Quantification of spheres was done by measuring the diameter of the spheres from random fields. Data represents mean ± S.E.M. (*N* = 3)

Because there was such a difference among cell lines in the proportion of ALDH vs SSTR1, we determined if the growth properties of these cells differed *in vitro*. In the CRC lines tested, the proportion of ALDH+ cells inversely correlates with proportion of SSTR1+ cells and with rate of proliferation. For example, HT29 cells (having a low ALDH/SSTR1 quotient) had the fastest growth rate, while the SW480 cells (having a high ALDH/SSTR1 quotient) had the slowest growth rate (Fig. 3a). Indeed, the doubling time for these two cell lines was 2.5 days for the HT29 cells and 4 days for the SW480 cells. Thus, the proliferation rate appears to inversely correlate with the ALDH/SSTR1 quotient.

## HT29 and SW480 CRC cell lines possess different degrees of self-renewal based on sphere-forming ability

One important characteristic of SCs is their ability to self-renew and give rise to more SCs or progenitor cells. From the data just presented, HT29 cells contain a low percentage of stem-like ALDH+ cells and high percentage of SSTR1+ cells, while the SW480 cell line contains a higher percentage of ALDH+ cells, but lower percentage of SSTR1+ cells (Fig. 1a). To test these cells for the SC property of self-renewal we evaluated their sphere-forming ability. When grown in soft agar assays, the SW480 cells gave rise to spheres that were significantly smaller in size than spheres that grew from the HT29 cells which were significantly larger in size (Fig. 3b). Thus, similar to the proliferation rate, the sphere-forming ability appears to inversely correlate with the ALDH/SSTR1 quotient.

## Treatment of CRC cell lines with somatostatin

**Fig. 4:**
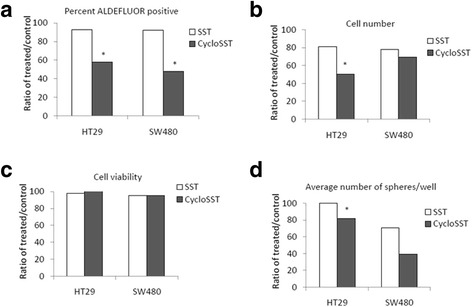
Somatostatin treatment on HT29 and SW480 cells does not change the ALDH+ population size or self-renewal abilities but inhibition of somatostatin decreases the ALDH+ population size. HT29 and SW480 cells were serum starved and then treated with 500 nM somatostatin or cyclosomatostatin for 48 h. Cells were trypsinized and analyzed for changes in ALDH positive cells, cell numbers, and cell viability. Somatostatin treatment did not significantly change the percentage of ALDH positive cells in either cell line for the number of ALDH positive cells (**a**), cell number (**b**), or cell viability (**c**), as compared to the control untreated cells. Cyclosomatostatin decreased the percentage of ALDH positive cells in both cells lines and only the cell number in HT29 cells, as compared to the controls. Data points represent the mean average values of treated cells over the controls (*N* = 3) * *p* ≤ 0.05. HT29 and SW480 cells were plated in ultra low attachment 6-well plates for colonosphere assay comparing untreated cells to somatostatin and cyclosomatostatin treatments, After 10 days, the number of spheres per well were counted and average numbers per well were calculated. Data points represent the mean average values of treated cells over the controls (**d**). SST = Somatostatin and CycloSST = Cyclosomatostatin

Because our results showed that the cell lines express SSTR1 and have different growth properties, we also wanted to test for their responsiveness to activating ligand. Accordingly, HT29 and SW480 cells were treated with somatostatin (SST). We first did a dose response curve of SST for each cell line (Additional file 2: Figure S2). The cell lines were then treated with exogenous somatostatin for 48 h and changes in ALDH+ cells, total cell numbers, cell viability and sphere formation were analyzed. The SST treatment decreased cell proliferation ~20% in both HT29 and SW480 (Fig. 4b). The HT29 cells exhibited no significant changes in ALDH+ cells, as analyzed by ALDH activity, as well as, no significant changes in cell viability or sphere formation, as compared to the vehicle controls (Fig. 4a-d). Similarly, exogenous somatostatin did not significantly change the ALDH+ cells, cell viability or sphere formation in SW480 cells (Fig. 4 a-d). All values shown in the graphs represent the ratio of treated cells over the appropriate control. Overall, somatostatin treatment on the two CRC cell lines did not significantly change the ALDH+ population size or self-renewal ability.

## Treatment of CRC cell lines with cyclosomatostatin

We further wanted to see if competitive inhibition of somatostatin would change the percentage of ALDH+ cells in the CRC cells. The cell lines were then treated with exogenous cyclosomatostatin for 48 h and changes in ALDH+ cells, total cell numbers, cell viability and sphere formation were analyzed. The HT29 cells exhibited a significant decrease in ALDH+ cells, cell count (50%) and sphere formation upon inhibition of somatostatin signaling, without affecting cell viability (Fig. 4a-d. Addition of the somatostatin inhibitor to the SW480 cells caused a similar result with a significant decrease in the percentage of ALDH+ cells, a decrease in sphere formation and in cell count (30%), without affecting cell viability (Fig. 4 a-d). All values shown in the graphs represent the ratio of treated cells over the appropriate control.

## Co-culture of ALDH+ cells with SSTR1+ cells

To assess whether SSTR1+ cells might modulate the growth of ALDH+ cells, ALDEFLUOR+ and SSTR1+ cells were sorted from HT29 colon cancer cell line and plated in a 1:1 ratio and allowed to grow in normal growth medium. After one week in standard tissue culture, cells were assessed for degree of culture confluence. And cells were trypsinized and analyzed for percent ALDEFLUOR+ cells. Results show that co-cultures of ALDH+ cells and SSTR1+ cells have reduced cell proliferation and an increased percentage of ALDEFLUOR positive cells (Additional file 4: Figure S4).

**Fig. 5:**
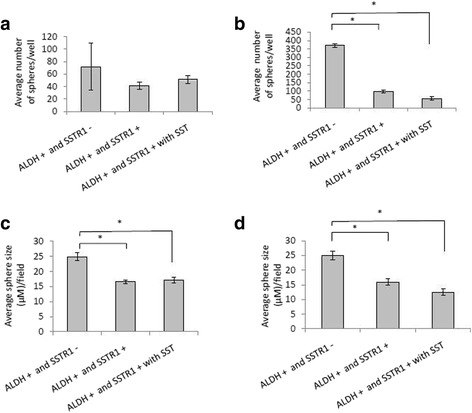
SSTR1 cell signaling limits sphere formation and cell proliferation of ALDH positive cells. ALDH positive cells were cultured in low attachment dishes for colonosphere assay under various conditions with or without exogenous SST for HT29 (**a** and **c**) and SW480 (**b** and **d**) cells. These experiments were all done in duplicate, * *p* ≤ 0.05. SST = Somatostatin and CycloSST = Cyclosomatostatin

Next, we wanted to see if there is a paracrine effect of SSTR1+ cells on ALDH+ cells. Both cell types were sorted from HT29 and SW480 lines and co-cultured under different conditions in ultra-low attachment dishes with transwell inserts (0.4 um). ALDH+ cells grown in the presence of signaling via SSTR1+ cells (with or without exogenous somatostatin) significantly decreased the size of spheres formed from the HT29 cells (Fig. 5a and c). Additionally, co-cultures of ALDH+ and SSTR1+ cells from the SW480 line showed a significant decrease in both the number and sizes of spheres (Fig. 5b and d). Thus, based on sphere formation, it appears that there is a paracrine effect of SSTR1+ cells on ALDH+ cells from HT29 and SW480 cell lines.

## Discussion

A more complete understanding of the effect of colonic NECs on the SC population in the normal colon is crucial to understanding SC function and cell proliferation and will likely provide insight into the mechanism underlying SC overpopulation that drives CRC growth. It is known that somatostatin and SSTR expression is found in the normal colon and SSTR1+ cells are located in close proximity to SCs within the crypt SC niche (Additional file 1: Figure S1) [5, 21]. Being in close proximity to each other, we surmised that the interactions and communication between these cell types may be crucial to regulation of normal SCs. Research has shown that the SCs of the colonic crypt are relatively quiescent as SC do not divide as rapidly as other cells in the epithelium and dysregulation of the crypt homeostasis is what leads to CRC development [2, 22]. Focus has been placed on different molecular factors involved in colonic SC regulation such as microRNAs [23], transcription factors [24], and growth factors [25] that can regulate SC function. However, we suggest that in addition to such molecular factors, attention should be placed on cellular mechanisms such as the cell-cell interactions between NECs and SCs.

In our study, we first looked at the expression of ALDH and SSTR1 in different CRC cell lines, as well as, in matched normal and tumor tissue samples (Fig. 1). In addition, we found that SSTR1+ cells from HT29 and SW480 cell lines also express other neuroendocrine markers (Additional file 5: Figure S5). We observed differential expression of ALDH and SSTR1 in the CRC cell lines and were able to identify that in fact these markers identify two separate subpopulations of cells found in human tissue samples and in CRC cell lines. Next, we showed expression of mRNA encoding SST and its receptors is present in matched normal and tumor tissue samples (Fig. 2). Interestingly, SST was expressed in all the normal tissue samples, but not expressed in the matched tumor tissues. When we analyzed additional normal tissue samples (ones without matched tumor), the results still showed presence of SST expression in all samples analyzed (data not shown). In addition, expression of SSTR1 was present in both primary normal and matched tumor colon samples, as well as, in the normal only tissue samples analyzed (data not shown). SST receptors have also been reported to be differentially expressed in various tumor types [26], and our data from different CRC tissue samples supports the expression of SSTR1 [27].

Next, the expression of SST mRNA and its receptors was analyzed in the SW480 and HT29 CRC cell lines. The results showed a trend similar to the pattern of expression in normal and tumor tissue samples. The HT29 cells, which were originally derived from a low grade, well-differentiated tumor, expressed SST and all five of its receptors, but the SW480 cells, which were originally derived from a high grade undifferentiated tumor, did not express SST or SSTR5. Of note, at the protein level, SSTR1+ cells were expressed in all CRC cell lines and normal and tumor colon tissue samples. That SST and SSTR1 were always expressed in the normal colonic epithelial cells, and SST was completely lost in the tumor cells while SSTR1 was also expressed in the tumor cells of some patients is an important finding. Interestingly, a recent study similarly reported that SST expression is lost in CRC yet expressed in the normal colonic epithelium, thus supporting our findings [28].

Along with identifying the presence of ALDH+ and SSTR1+ subpopulations in the CRC cell lines and human tissue samples, we tested the growth abilities of HT29 and SW480 cell lines in terms of cell proliferation and self-renewal. A major aim of this study was to study the “stemness” characteristics of the HT29 and SW480 cells relative to the proportion of ALDH+ and SSTR1+ cells. Accordingly, several CRC cell lines were screened that were originally derived from CRCs having differences in histological grade, stage and differentiation. Within the CRC cell lines, we predicted that the relative proportion of ALDH and SSTR1+ cells in any given cell line will correlate with a certain rate of cell proliferation and self-renewal. Therefore, the CRC cell line that has a higher proportion of ALDH is predicted to show more characteristics of “stemness” (slower proliferation and smaller spheres). First, we obtained the cell proliferation rate of various cell lines over a five day time course. Our data on the *in vitro* growth of the cell lines showed that the proliferation rate inversely correlates with ALDH/SSTR1 quotient (Fig. 3). For example, HT29 cells (having a low ALDH/SSTR1 quotient) had the fastest growth rate (doubling time of 2.5d), while the SW480 cells (having a high ALDH/SSTR1 quotient) the slowest growth rate (doubling time of 4d).

The next step was to assess the ability to self-renew and form spheres from single cells embedded in soft agar. Our study showed the HT29 cells with a lower proportion of ALDH+ cells formed fewer but larger sized spheres than the SW480 cells (Fig. 3). This could be due to the fact that sphere formation assays measure the ability of a single SC to form many cells in culture [20, 29] so the more SCs in a culture the more spheres are formed. However, the difference between the sizes of the spheres could be due to the proliferation rate of the cell lines and the non-SC daughters with a faster doubling time could give rise to larger sized spheres. Our data indicates that the cell line with relatively more ALDH cells (SW480) showed more features of true quiescent- like SCs in terms of slower cell proliferation and smaller-sized spheres. On the other hand, the cell line with fewer ALDH+ cells (HT29) showed more features of transit amplifying or progenitor cells with a significantly higher proliferation rate and large-sized spheres.

That each CRC cell line has a unique ALDH+/SSTR1+ ratio that is maintained constant over multiple passages and that correlates with its growth dynamics in terms of proliferation and sphere-forming ability, suggests feedback mechanisms exist between ALDH+ and SSTR1+ cells that contribute to regulation of the ALDH+ population size.

To further explore the role of SST and its signaling via SSTR1 receptor, we chose to treat HT29 and SW480 cell lines with exogenous SST with the goal of understanding the effect on the ALDH population size. Already knowing the baseline proportions of ALDH+ to SSTR1+ cells, we could feasibly measure the changes to the ALDH+ population due to enhanced SSTR1 signaling. SST is a known to have anti-proliferative effects in normal dividing cells like intestinal mucosal [5] yet the mechanism involved is not well understood. SST can bind to all five SST receptors subtypes and exert its effects of anti-proliferation or secretion inhibition [5, 30, 31]. The dose of SST was determined using a concentration range of SST that is close to physiological levels. In treatment of both HT29 and SW480 cell lines with exogenous SST, it did not affect the ALDH population size in either line (Fig. 4). The fact that SST treatments did not change the ALDH population size or viability while decreasing proliferation (Fig. 4a-c) could be a result of prolonged cell cycle progression or delayed maturation.

On the other hand, the addition of cyclosomatostatin, a somatostatin receptor antagonist, to the SW480 and HT29 cells showed that when SST signaling was blocked, there was a significant decrease in the ALDH+ population in both cell lines. There was also a decrease in cell number after treatment, but no loss in cell viability. Given that upon inhibition of SSTR1 signaling, there is a significant decrease in the ALDH+ cell numbers, it seems that an appropriate level of SST and SSTR1 signaling is needed to maintain the ALDH+ cells in a slower cycling state and any more or less SSTR1 signaling can cause a significant change in the ALDH+ population size. Thus, results from both SST and cycloSST treatment suggests that just the right amount of SST signaling is necessary for maintenance of ALDH+ cells and generation of its proliferative progeny cell population.

Another important function of NECs in general is their ability to signal through paracrine or autocrine mechanisms [26]. To determine if SSTR1+ cell regulation of the ALDH+ cells (Additional file 4: Figure S4) might occur via paracrine signaling, we designed experiments in which ALDH+ and SSTR1+ cells were co-cultured in transwell dishes to avoid their direct cell-to-cell contact. In the HT29 cells, there were a reduced number of spheres formed and a significant difference in the average sizes, as compared to the co-cultures of ALDH+ cells with SSTR1 negative cells (Fig. 5a and c). In the SW480 cells, there was a significant decrease in the size and number of spheres formed between the ALDH+ and SSTR1 negative cells, and ALDH+ and SSTR1+ cells (Fig. 5b and d).

HT29 cells are fast growing colon cancer cells that contain few ALDH+ cells and more differentiated cell types. In comparison, SW480 cells are more undifferentiated and have a slower growth rate. Based on our data, co-culturing of ALDH+ cells with SSTR1+ cells had different effects depending on the cell line studied. Since HT29 cells have a faster growth rate (with a doubling time of less than 24 h), the effect of the SSTR1+ cells on ALDH+ cells was minimal. On the other hand, the effect of SSTR1+ cells on ALDH+ cells from the SW480 line exhibit a significant reduction in both sphere size and number. SSTR1+ cells affected the growth of the ALDH+ cells by limiting the number of spheres and size of spheres formed.

Collectively, our results indicate that SSTR1+ cells have a paracrine signaling type of interaction with colonic ALDH+ cells. Through our co-culture studies, there appears to be paracrine-mediated regulation via SSTR1+ cells to maintain the ALDH population size in a state of quiescence, leading to slower cell cycling. Since ALDH+ cells lack SST and SSTR1 expression and the ALDH negative cell population cells expressed both SST and SSTR1, we conjecture that SST signaling auto-regulates the rate of NEC maturation in a feedback manner as SCs mature along the NEC lineage, which contributes to quiescence of SCs and inhibition of proliferation.

## Conclusion

In summary, this study identifies a new role for SSTR1 cell signaling in regulation of colonic ALDH+ cells. The fact that the CRC cell lines each maintain a unique proportion of ALDH+ and SSTR1+ cells over time, we surmised these proportions are maintained constant through feedback mechanisms involving ALDH and SSTR1 cell subpopulations. Given that both SST and cycloSST produce growth suppressive effects suggests that SST signaling modulates this feedback mechanism. Moreover, the ability of SSTR1+ cells to decrease sphere formation and proliferation of ALDH+ cells in transwell cultures indicates that the ALDH subpopulation is regulated by SSTR1 via a paracrine mechanism. Since ALDH+ cells lack SST and SSTR1 expression, we conjecture that SST signaling controls the rate of SSTR1 NEC maturation as ALDH+ cells mature along the NEC lineage, which contributes to quiescence of ALDH+ cells and inhibition of proliferation. Our findings may have clinical significance since modulating the quiescent state of SCs could make them more susceptible to certain SC-targeted therapeutics designed to either differentiate SCs or induce their apoptosis.

## Additional files

Additional file 1:
**Figure S1.** Representative images of normal human colonic crypts to show positive ALDH1 and SSTR1cells. These images are a representative immunostaining of patient normal colon tissue sections that stained positive for ALDH1 (1:100, BD Biosciences) and SSTR1 (1:100, Advanced Targeting System). Both of these cell markers are expressed in the normal colonic crypts. Images taken on the Zeiss Epi-Fluorescence microscope or Zeiss 780 Confocal microscope and using the 20x objective. (JPG 22 kb)
Additional file 2:
**Figure S2.** Dose response curves for concentrations of somatostatin in HT29 and SW480 cells. This data is based on the counting of HT29 cells and SW480 cells after 48 h of treatment. Cells were plated at 250,000 cells/well of a 6-well dish, allowed to attach overnight and then serum starved for 24 h. Increased concentrations of somatostatin were added to the cells and after 48 h cells were trypsinized and counted. All cell counts were greater than 95% viable, which was determined by trypan blue exclusion on the cell counter. Experiment was done in triplicate and error bars represent ± SEM. (JPG 22 kb)
Additional file 3:
**Figure S3.** Expression of ALDH positive cells via ALDEFLUOR assay in SW480 and HT29 colon cancer cell lines. (A) The result of the ALDEFLUOR assay performed on HT29 cells and imaged on the Zeiss Epi-Fluorescence microscope before analyzed on the flow cytometer. (B) The results of the ALDEFLUOR assay performed on SW480 cells and imaged on the Zeiss Epi-Fluorescence microscope before analyzed on the flow cytometer. Image was enhanced to show brightness of green color. Each panel set shows the FITC channel for ALDEFLUOR positive cells and a bright field image of all the cells. (JPG 28 kb)
Additional file 4:
**Figure S4.** Co-cultures of ALDH+ cells and SSTR1+ cells show decreased cell proliferation and an increased percentage of ALDEFLUOR positive cells. ALDEFLUOR+ and SSTR1+ cells were sorted from the HT29 colon cancer cell line and plated in a 6-well culture dish at a 1:1 ratio of each cell type and allowed to grow in normal growth medium. Culture medium was changed every two days. Representative images were taken after 7 days of co-culture (A) and then trypsinized and analyzed for percent ALDEFLUOR positive (B). ALDH+ cells grown with SSTR1+ cells limit the growth of the stem cells and keeps the cells more stem-like due to increased percentage of ALDEFLUOR positive cells. (JPG 33 kb)
Additional file 5:
**Figure S5.** Differential expression of neuroendocrine markers in SSTR1+ cells from HT29 and SW480 cell lines. SSTR1+ cells were sorted from the HT29 and SW480 colon cancer cell lines and RNA was isolated. Conventional RT-PCR was performed on these samples to show expression of other neuroendocrine markers co-expressed in the sorted neuroendocrine cells, SSTR1+. In both cell lines, the SSTR1+ cell population expresses the broad, widely accepted marker for neuroendrocrine cells, CgA. The other neuroendorcrine markers are differentially expressed between the two cell lines, as seen in the image. CgA = Chromogranin A, NSE = enolase 2, NCAM = neural cell adhesion molecule. (JPG 15 kb)

## Abbreviations

ALDH: Aldehyde dehydrogenase
CRC: Colorectal cancer
CSC: Cancer stem cell
CycloSST: Cyclosomatostatin
NECs: Neuroendocrine cells
SC: Stem cells
SST: Somatostatin
SSTR1: Somatostatin receptor type 1
