# Supplementary figures and images for: Somatostatin signaling via SSTR1 contributes to the quiescence of colon cancer stem cells
Source: BMC Cancer. 2016 Dec 7;16:941. doi: 10.1186/s12885-016-2969-7 (PMC5142402; doi:10.1186/s12885-016-2969-7)

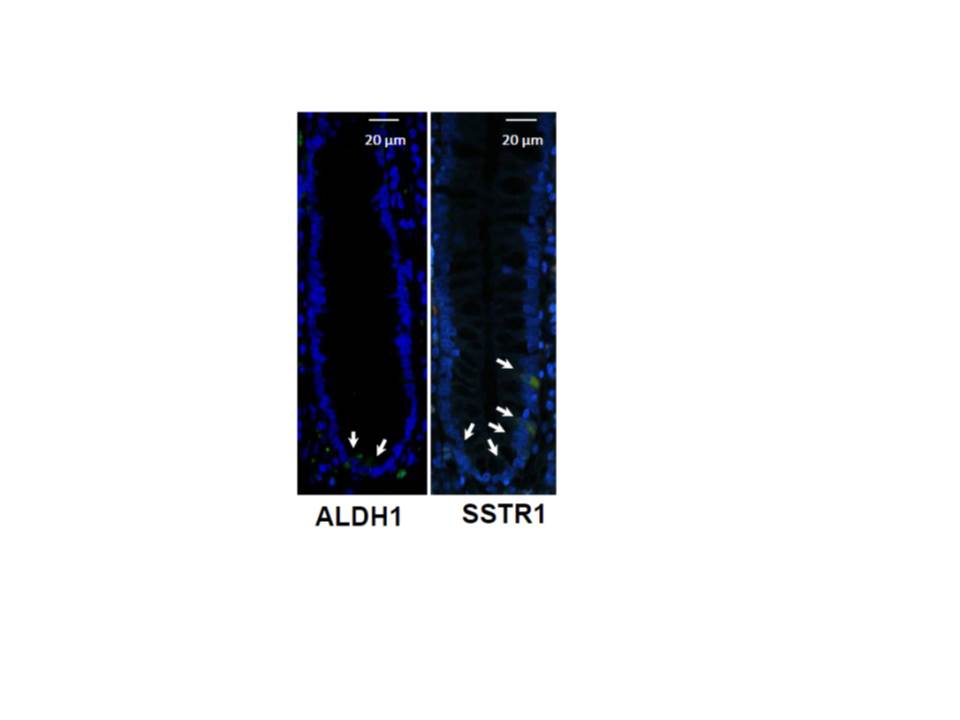

Supplement: Additional file 1: — Figure S1. Representative images of normal human colonic crypts to show positive ALDH1 and SSTR1cells. These images are a representative immunostaining of patient normal colon tissue sections that stained positive for ALDH1 (1:100, BD Biosciences) and SSTR1 (1:100, Advanced Targeting System). Both of these cell markers are expressed in the normal colonic crypts. Images taken on the Zeiss Epi-Fluorescence microscope or Zeiss 780 Confocal microscope and using the 20x objective. (JPG 22 kb) [file 12885_2016_2969_MOESM1_ESM.jpg]

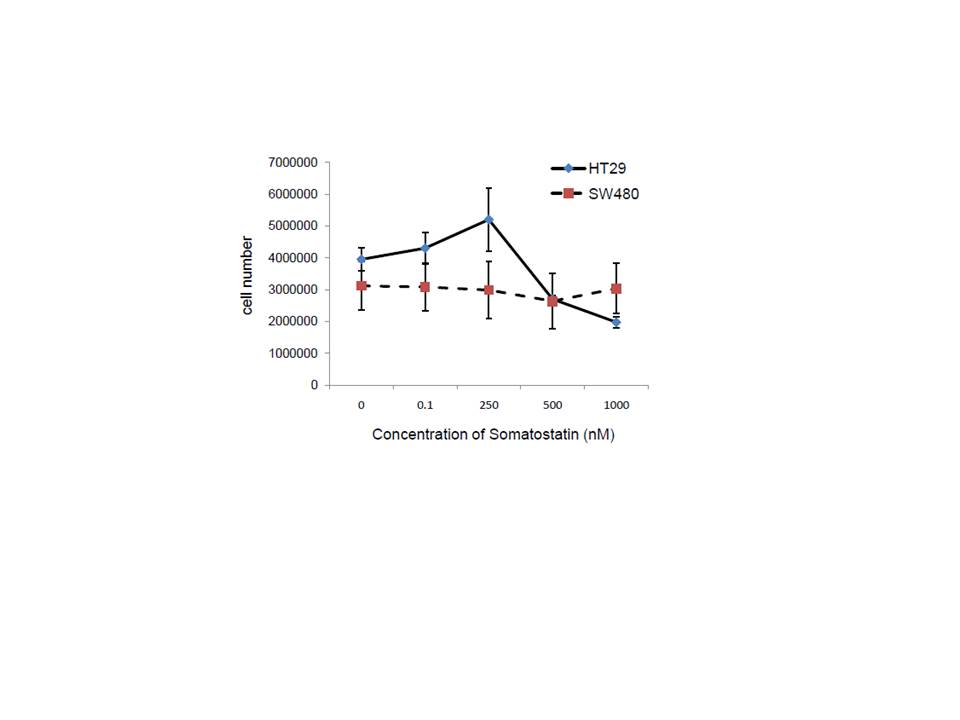

Supplement: Additional file 2: — Figure S2. Dose response curves for concentrations of somatostatin in HT29 and SW480 cells. This data is based on the counting of HT29 cells and SW480 cells after 48 h of treatment. Cells were plated at 250,000 cells/well of a 6-well dish, allowed to attach overnight and then serum starved for 24 h. Increased concentrations of somatostatin were added to the cells and after 48 h cells were trypsinized and counted. All cell counts were greater than 95% viable, which was determined by trypan blue exclusion on the cell counter. Experiment was done in triplicate and error bars represent ± SEM. (JPG 22 kb) [file 12885_2016_2969_MOESM2_ESM.jpg]

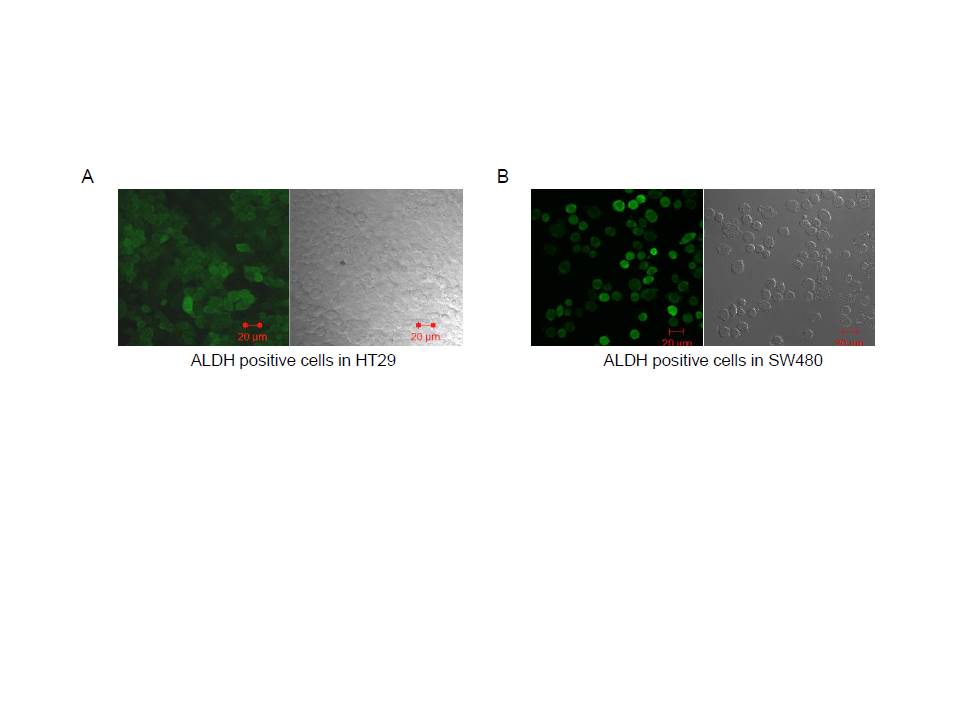

Supplement: Additional file 3: — Figure S3. Expression of ALDH positive cells via ALDEFLUOR assay in SW480 and HT29 colon cancer cell lines. (A) The result of the ALDEFLUOR assay performed on HT29 cells and imaged on the Zeiss Epi-Fluorescence microscope before analyzed on the flow cytometer. (B) The results of the ALDEFLUOR assay performed on SW480 cells and imaged on the Zeiss Epi-Fluorescence microscope before analyzed on the flow cytometer. Image was enhanced to show brightness of green color. Each panel set shows the FITC channel for ALDEFLUOR positive cells and a bright field image of all the cells. (JPG 28 kb) [file 12885_2016_2969_MOESM3_ESM.jpg]

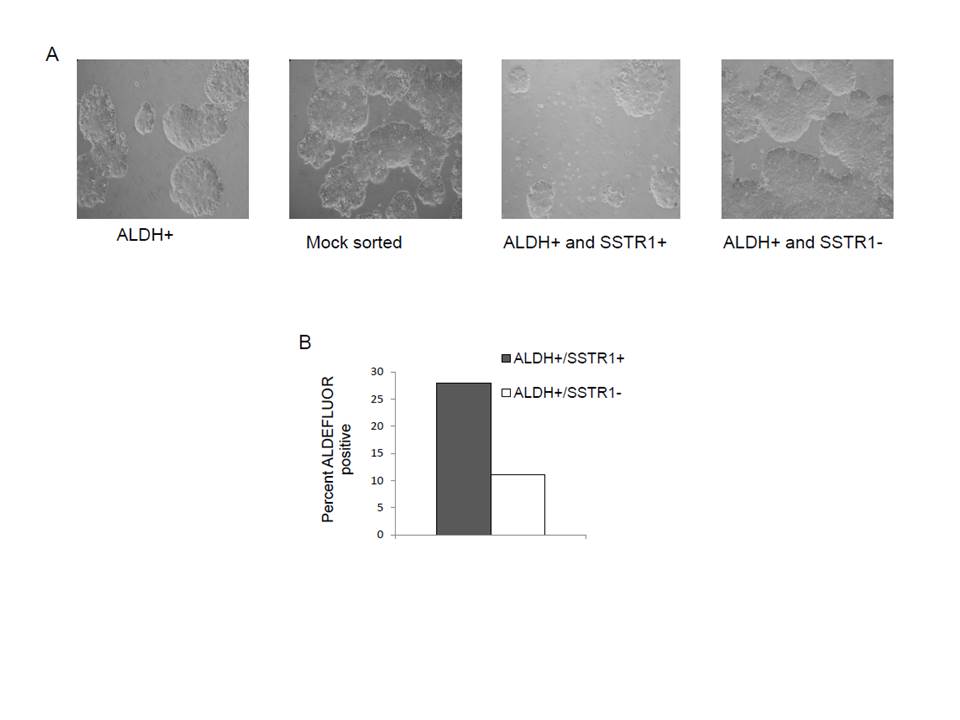

Supplement: Additional file 4: — Figure S4. Co-cultures of ALDH+ cells and SSTR1+ cells show decreased cell proliferation and an increased percentage of ALDEFLUOR positive cells. ALDEFLUOR+ and SSTR1+ cells were sorted from the HT29 colon cancer cell line and plated in a 6-well culture dish at a 1:1 ratio of each cell type and allowed to grow in normal growth medium. Culture medium was changed every two days. Representative images were taken after 7 days of co-culture (A) and then trypsinized and analyzed for percent ALDEFLUOR positive (B). ALDH+ cells grown with SSTR1+ cells limit the growth of the stem cells and keeps the cells more stem-like due to increased percentage of ALDEFLUOR positive cells. (JPG 33 kb) [file 12885_2016_2969_MOESM4_ESM.jpg]

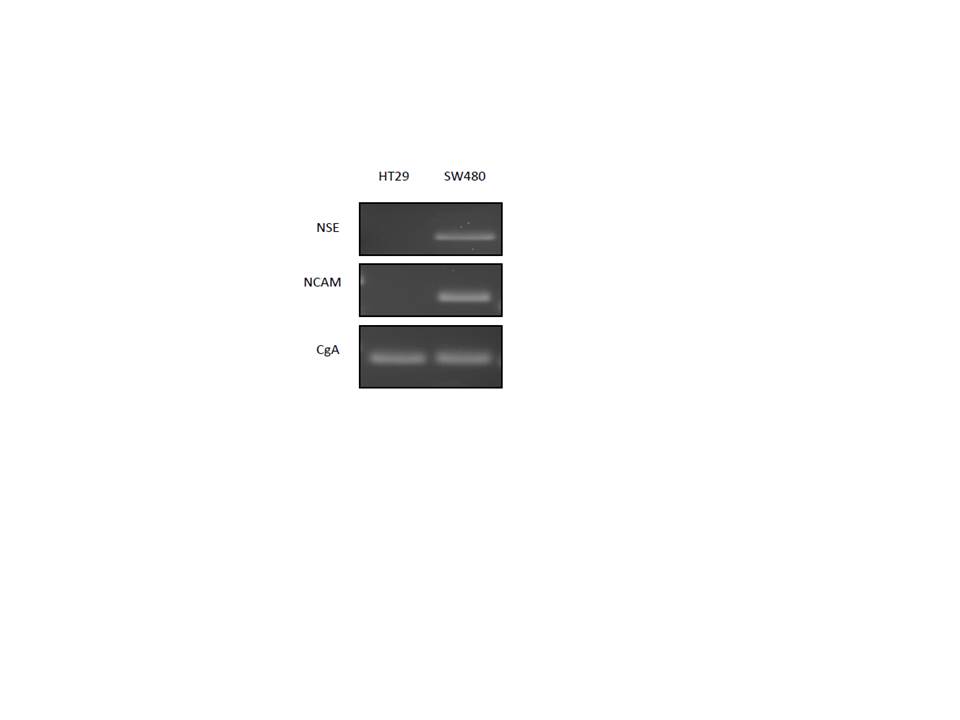

Supplement: Additional file 5: — Figure S5. Differential expression of neuroendocrine markers in SSTR1+ cells from HT29 and SW480 cell lines. SSTR1+ cells were sorted from the HT29 and SW480 colon cancer cell lines and RNA was isolated. Conventional RT-PCR was performed on these samples to show expression of other neuroendocrine markers co-expressed in the sorted neuroendocrine cells, SSTR1+. In both cell lines, the SSTR1+ cell population expresses the broad, widely accepted marker for neuroendrocrine cells, CgA. The other neuroendorcrine markers are differentially expressed between the two cell lines, as seen in the image. CgA = Chromogranin A, NSE = enolase 2, NCAM = neural cell adhesion molecule. (JPG 15 kb) [file 12885_2016_2969_MOESM5_ESM.jpg]
